# Effect of point of care information on inpatient management of bronchiolitis

**DOI:** 10.1186/1471-2431-7-4

**Published:** 2007-01-24

**Authors:** W James King, Nicole Le Saux, Margaret Sampson, Isabelle Gaboury, Mark Norris, David Moher

**Affiliations:** 1 Department of Pediatrics, University of Ottawa, Ottawa, Ontario, Canada; 2 Chalmers Research Group, Children's Hospital of Eastern Ontario Research Institute, Ottawa, Ontario, Canada; 3 Division of Pediatric Medicine, Children's Hospital of Eastern Ontario, 401 Smyth Road, Ottawa, Ontario, K1H 8L1, Canada

## Abstract

**Background:**

We studied the effects of access to point-of-care medical evidence in a computerised physician order entry system (CPOE) on management and clinical outcome of children with bronchiolitis.

**Methods:**

This was a before-after study that took place in a Canadian tertiary care paediatric teaching hospital. The intervention was a clinical evidence module (CEM) for bronchiolitis management, adapted from Clinical Evidence (BMJ Publishing Group) and integrated into the hospital CPOE. CPOE users were medical trainees under the supervision of staff physicians working in the infant ward. Use of antibiotics, bronchodilators and corticosteroids; disease severity; length of hospital admission; and trainee use and perception of the CEM were measured before and after CEM introduction.

**Results:**

334 paediatric inpatients age 2 weeks to 2 years, with a clinical diagnosis of bronchiolitis; 147 children the year preceding and 187 children the year following introduction of a Clinical Evidence Module (CEM). The percentage of patients receiving antibiotics fell from 35% to 22% (relative decrease 37%) following the introduction of the CEM (p = 0.016). Bronchodilator use was high but following the CEM patients no longer received more than one variety. Steroid usage and length of hospitalisation were low and unaffected. Trainees found the CEM to be educational.

**Conclusion:**

Readily accessible clinical evidence at the point of care was associated with a significant decrease in antibiotic use and an end to multiple bronchodilator use. The majority of physician trainees found the CEM to be a useful educational tool.

## Background

A computerised clinical decision support system (CDSS) may improve the efficiency and efficacy of patient care, in part by facilitating incorporation of clinical evidence into therapeutic decision-making. As with all health care innovations, CDSSs require rigorous evaluation before their widespread use in patient care[1]. A systematic review of 68 controlled trials found improvements in health care practitioner performance with 66% of the innovations, principally in drug dosing and preventive care for treatment of hypertension, diabetes and acquired immunodeficiency syndrome[2]. Another, more recent systematic review found that successful CDSSs are computerised, provide decision support as part of clinician workflow, provide recommendations rather than just assessments, and provide decision support at the time and location of decision making[3].

Bronchiolitis was chosen as the context for evaluation of a computer-based CDSS providing medical evidence at the point of care. This virus-induced acute inflammation of the lower respiratory tract is the most common reason for hospitalisation of infants. Over 100,000 children under 1 year of age are hospitalised annually in North America, with an estimated annual health care cost of more than $300,000,000[4]. Bronchiolitis treatment is largely supportive, although therapies (bronchodilators, systemic steroids, and antibiotics) may benefit some patients. Evidence-based clinical practice guidelines have modified care and improved clinical outcome[5-7].

Despite these guidelines, published studies of bronchiolitis treatment often contradict each other, and may be contrary to local management strategies. For instance, supra-infection with bacteria in uncomplicated cases of bronchiolitis is uncommon and the recommendation has been not to routinely use antimicrobials. However, antibiotics are commonly given to many hospitalised infants[8,9]. Needless antimicrobial therapy has significant care implications, because antibiotics confer potential risks of infection with resistant bacteria, as well as adverse drug reactions. Successful implementation of a clinical evidence CDSS depends upon high quality credible information, ready access within existing tasks, upkeep of the information as research evolves and assessment of physician actions and patient outcomes. These elements were incorporated into this study, wherein point-of-care access to up-to-date clinical evidence regarding bronchiolitis management in hospitalised children was provided through a computerized physician order entry system (CPOE) to medical students and residents.

## Methods

### Study design, setting

A quasi experimental before-after study conducted at the Children's Hospital of Eastern Ontario (CHEO), a tertiary care paediatric hospital in Ottawa, Canada. The CHEO Research Ethics Committee approved the study.

### Study population

Bronchiolitis patients on CHEO's infant ward were identified by discharge diagnosis ICD-9-CM codes (ICD-9 codes:466.11, 466.19 and 480.1)[10] during peak bronchiolitis periods: pre-intervention November 01, 2000 – March 31, 2001; and intervention November 01, 2001 – March 31, 2002. Participants were included if they were age 2 weeks to 2 years. Patients with a pre-existing diagnosis of asthma or patients with wheezing and/or cough who had previously been treated with bronchodilators or steroids were excluded.

Residents and medical students from the University of Ottawa entered all orders, under the supervision of the attending staff doctor.

### Intervention

A clinical evidence module (CEM) was integrated into the Medical Logic Module of CHEO's CPOE system (Sunrise Clinical Manager™, Eclipsys Corporation, Boca Raton, FL).

The CEM was based on the information in "Clinical Evidence", a monthly, updated review of evidence on the effects of common clinical interventions, used with permission from the BMJ Publishing Group[11]. Electronic searches of Medline were also used to monitor the medical literature during the intervention period, but no changes were made to the CEM.

**Figure 1:**
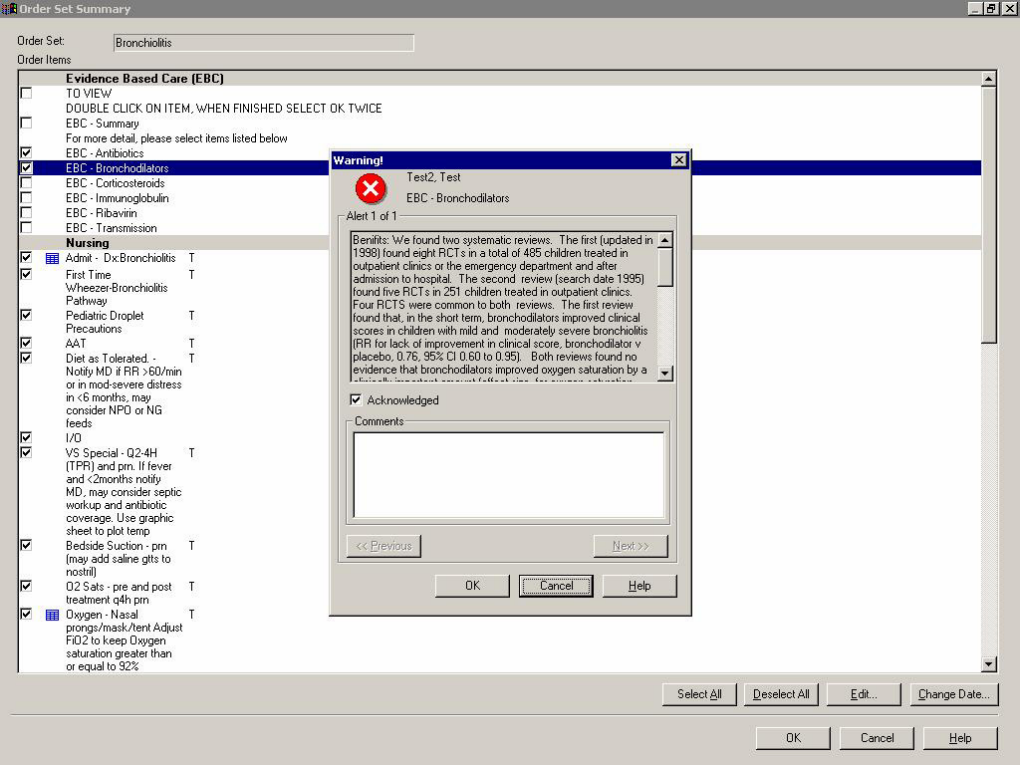
**Screen Shot of CEM and Bronchiolitis Order Entry Set**. (Used with permission of Eclipsys Corporation, Boca Raton, FL).

The CEM was available when trainees and physicians on CHEO's infant ward selected the bronchiolitis order entry set. The top quarter of the screen was entitled "Evidence Based Care", with options to view evidence regarding: antibiotics; bronchodilators; corticosteroids; ribavirin; immunoglobulins; and nursing interventions (Figure 1). The user could access a "bottom line" summary of the evidence as well as more complete syntheses for individual topics, and integrate the evidence as deemed clinically suitable. Physicians and trainees were informed by e-mails and reminded at monthly staff meetings that the CEM was available. They were not informed that management of bronchiolitis patients would be monitored, in order to minimise the potential impact of that measurement on their practice.

### Outcome measures

The primary outcome measures were the frequency of ordering of antibiotics, bronchodilators and corticosteroids. Ribavirin, immunoglobulin and nursing interventions were not included as outcome measures *a priori*, as existing hospital guidelines governed the use of these interventions.

The secondary outcome measure was the length of stay in hospital (LOS).

In order to compare the severity of patients' illness, as well as costs of hospitalisation, the resource intensity weighting (RIW) of each hospitalisation was calculated[12]. The RIW is a standard administrative factor generated based on discharge diagnoses and hospitalisation chart review.

Antibiotic use for patients on the infant study ward who did not have a diagnosis of bronchiolitis and antibiotic use for all patients in hospital during the study period were tabulated.

Outcome measures were extracted electronically from hospital pharmacy and discharge records.

### Statistical analysis

Age and illness severity were compared between study groups using Wilcoxon rank sum tests, and gender was compared using Fisher's exact test. Due to a significant difference in age between pre- and post-intervention groups, all subsequent analyses were adjusted for child's age within the regression model. Differences in the use of antibiotics, bronchodilators and steroids were assessed using Chi-square or Fisher's exact tests. Number of doses of those three principal medications were analysed using Poisson regression models. Cox regression models were fit to assess a difference in both length of stay in the hospital and length of stay on the floor. All reported p-values are two-sided and were declared statistically significant when they reached a 0.05 probability level.

### Post-intervention feedback

A 12-question survey of students and residents addressed utilisation, usefulness, potential improvements and general applicability of the CEM (see Additional 1).

## Results

A total of 334 children with a median age of 0.41 years (range: 0.02, 1.99) participated in this study; 147 children the year preceding and 187 children the year following the introduction of the CEM. There were no differences between the study groups with respect to gender or illness severity, as measured by the RIW. However, the group following introduction of the CEM were younger (p = 0.021) (Table 1).

The proportion of patients receiving antibiotics fell from 35% to 22% (relative decrease of 37%) following the introduction of the CEM (p = 0.019) (Table 2). At the same time, there was no significant difference in antibiotic use for other patients on the infant ward (originally estimated to 49.4% followed by a relative increase of 4.5%, p = 0.541). Similarly, no significant change was detected in the total population of hospitalized patients receiving antibiotics (originally 45.9% with a relative decrease of 7.0%, p = 0.265).

Bronchodilator use was universally high (94%) prior to and following the introduction of the CEM, but physicians had less variation in bronchodilator medications prescribed. Salbutamol was preferred over epinephrine before the introduction of the CEM, while preference was reversed when clinical evidence was provided (Table 2).

Corticosteroid use was uniformly low prior to (14%) and following (13%) the intervention (Table 2).

Median length of stay (LOS), from the time of registration in the emergency department to discharge from the hospital, increased from 2.8 to 2.9 days following introduction of the CEM (p = 0.125). Median LOS on the floor was unchanged prior to and following introduction of the CEM (2.8 days).

Fifty surveys were returned (90% response rate) from medical trainees; 19 (38%) residents and 31 (62%) medical students. Although 94% of respondents had admitted a patient with bronchiolitis, 55% stated that they were unaware of the CEM availability. Medical students (19%) were less likely and paediatric residents (77%) more likely to have reviewed the CEM. Of respondents who had read the CEM, all medical students reported that the review was clinically helpful while 29% of the senior pediatric residents reported that the information was not of assistance. All respondents agreed that the CEM had been educational and that point of care evidence would have merit if expanded to other clinical conditions.

## Discussion

In an increasingly complex medical environment, decision support systems are promising tools for clinicians, overloaded with information. While a CDSS enables the delivery of evidence-based practice at the point of care, considerable challenges exist for CDSS to support the management of complex and multiple medical conditions[13]. In this study Bronchiolitis provided a context to evaluate a computer-based CDSS wherein clinical evidence was made readily available on a commercial CPOE system. There is evidence that implementing bronchiolitis clinical practice guidelines at the point of care decreases unwarranted treatment variation[7,8,14]. Also, selected CDSSs have been able to improve physician performance and substantially reduce medication error rates, though the actual impact on patient outcomes is unknown[3,15].

Evaluating the effectiveness of specific elements of a CDSS and its delivery via a commercial CPOE system on a busy inpatient unit revealed that the clinical management of infants hospitalised with bronchiolitis was improved. There was a decrease in the proportion of patients receiving antibiotics and while a large proportion of patients continued to receive bronchodilators, fewer patients received multiple bronchodilators. The current *Clinical Evidence*[16] does not support the routine use of bronchodilators for hospitalised infants, however, the CEM used in this study quoted an older version of *Clinical Evidence *that recommended a trial dose of a bronchodilator, and to discontinue this therapy if there is no response[11]. Perhaps, this accounted for the decrease in the variation of the bronchodilators prescribed. Steroid use was low, and LOS in the ward was consistently < 3 days for the medians of the populations. They remained unchanged.

The limitations of this study highlight difficulties in the evaluation of interventions to improve complex systems of health care. For instance, the observational study design and lack of blinding are potential sources of bias. While the simple study design and straightforward outcome measures yielded clear results in terms of physicians' orders for antibiotics and bronchodilators, the study demonstrates associations between the use of the intervention, the observed outcomes and the process measures and does not prove a direct cause-and-effect relationship. Though we were not aware of any, other changes in bronchiolitis treatment patterns or antibiotic usage during the study period, such as other written or electronic guidelines, lectures, or handouts, may have influenced orders. However, for antibiotic usage we were able to demonstrate a decrease in usage specific to bronchiolitis while antibiotic use for other conditions and in other hospital units stayed the same or increased during the study. While many of the trainees entering orders were not aware of the intervention, this was primarily among medical students whose admitting orders are reviewed by senior pediatric residents, the majority of whom had reviewed the CEM. A difference in infants' age could potentially influence antibiotic use. However, fewer antibiotics were administered to the younger group, although physicians might be expected to be more likely to order antibiotics for younger infants[17]. A clinical order set for bronchiolitis was developed and incorporated into the CPOE system two years prior to the initiation of the study, so ready access to electronic order-sets was not a co-intervention. All treatment measurements were straightforward and exact, from computerised medical records, so the data were valid and reproducible.

Antibiotic over-use may have been addressed by other initiatives within the hospital or medical school. However, such a co-intervention would be expected to have a universal effect, and antibiotic use decreased only amongst the bronchiolitis patients. Although antibiotic use fell by 37%, a quarter of the patients continued to receive them. This compares favourably with reports of antibiotic administration to over half of bronchiolitis patients[9,18], but it would be desirable to decrease usage to as low a level as possible; antibiotic orders for only 9% of bronchiolitis patients has safely been achieved[7]. Finally, while this intervention had three of the four characteristics associated with success for CDSSs[3], it did not require acknowledgement or action as part of the work flow. This appears to be important, because about half of the medical trainees who encountered the CEM did not notice it.

Implementation and routine updating of computerized CDSS would require scarce resources allocated for patient care, so innovations must be evaluated to ensure that they are both economical and effective, to improve outcomes for patients and education of medical trainees. The implication of this study for clinicians and policymakers is that ready access to valid, relevant information empowers trainees to make evidence-based treatment choices, and may increase future use of evidence in clinical decision-making[19]. In this teaching hospital, the CEM served the dual role of improving clinical practice, and of imparting important information to medical trainees at an opportune moment. The majority of trainees, particularly those more junior in their training, found the provision of evidence at the point of care beneficial.

## Conclusion

In summary, provision of clinical evidence from a reputable source at the point of care influenced bronchiolitis treatment choices by medical trainees, at least in the areas of antibiotic and bronchodilator use. This model for providing information may be a helpful educational tool, as well as a means of improving patient health care management for other diseases. In the future we plan to study optimization of the operation, appearance and positioning in the computerised order entry process, and co-interventions to maximise utilisation.

## Abbreviations

CDSS – clinical decision support system;

CEM – clinical evidence module;

CHEO – Children's Hospital of Eastern Ontario;

CPOE – computerised physician order entry;

LOS – Length of stay;

RIW – resource intensity weighting

## Competing interests

The author(s) declare that they have no competing interests.

## Authors' contributions

WJK conceived of the study, participated in its design, coordinated the collection and acquisition the data, and drafted and revised the article for intellectual content. NLS participated in the design of the study, MS contributed to the literature review and the development and implementation of the evidence-based module, IG contributed to the analysis of the data and interpretation of the results, MN contributed to the design and coordination of the survey and DM contributed to the study design, development of the evidence-based module and the interpretation of the results. All of the authors made substantial revisions to the intellectual content of the manuscript and approved the final version.

## Pre-publication history

The pre-publication history for this paper can be accessed here:



## Figures and Tables

**Table 1:** Characteristics of Study Participants

**Characteristics**	**Pre-CEM introduction 2000 – 2001**	**Post-CEM introduction 2001 – 2002**	**p-value**
Age in years, median (range)	0.50 (0.03, 1.97)	0.36 (0.02, 1.99)	0.021
RIW, median (range)	0.63 (0.57, 3.94)	0.63 (0.63, 7.91)	0.186
Male, n (%)	83 (56.5)	113 (60.4)	0.502

**Table 2:** Antibiotic, Bronchodilator and Steroid use Prior to and Following CEM Introduction

	**Pre-CEM 2000 – 2001 n = 142**	**Post-CEM 2001 – 2002 n = 174**	**Difference (95% CI)**	**p-value**
**Antibiotic Prescription Patterns for Bronchiolitis Patients**				

Antibiotics, n (%)	49 (34.5)	38 (21.8)	12.7 (2.7, 22.5)	0.020
# antibiotics received:				
0, n (%)	93 (65.5)	136 (78.2)		
1, n (%)	28 (19.7)	22 (12.6)		
2, n (%)	16 (11.3)	12 (6.9)		
3, n (%)	4 (2.8)	3 (1.7)		
4, n (%)	1 (0.7)	1 (0.6)		
Median (range)	0 (0, 4)	0 (0, 4)	0 (0,0)	0.019

**Bronchodilator Prescription Patterns for Bronchiolitis Patients**				

Bronchodilators, n(%)	134 (94.4)	163 (93.7)	0.7 (-5.1, 6.1)	0.873
Salbutamol received, n (%)	102 (71.8)	77 (44.3)	27.6 (16.9, 37.4)	<0.001
Epinephrine received, n (%)	72 (50.7)	120 (69.0)	-18.3 (-28.6, -7.4)	0.005
Number of different bronchodilators received				
0 received, n (%)	8 (5.6)	11 (6.3)		
1 received, n (%)	57 (40.1)	64 (36.8)		
2 received, n (%)	37 (26,1)	37 (21.3)		
3 received, n (%)	18 (12.7)	34 (19.5)		
≥ 4 received, n (%)	22 (15.5)	28 (16.1)		
Median (range)	2 (0, 8)	2 (0, 9)	0 (0, 0)	0.637

**Steroid Prescription Patterns for Bronchiolitis Patients**				

Inhaled Steroids				
0, n (%)	132 (93.0)	167 (96.0)		
1, n (%)	10 (7.0)	6 (3.4)		
2, n (%)	0 (0.0)	1 (0.6)		
Median (range)	0 (0, 1)	0 (0, 2)	0 (0, 0)	0.510
Systemic Steroids				
0, n (%)	129 (90.8)	157 (90.2)		
1, n (%)	13 (9.2)	17 (9.8)		
Median (range)	0 (0, 1)	0 (0,1)	0 (0, 0)	0.629
**Total Inhaled and Systemic steroids**				
0, n (%)	122 (85.9)	152 (87.4)		
1, n (%)	17 (12.0)	19 (10.9)		
2, n (%)	3 (2.1)	3 (1.7)		
Median (range)	0 (0, 2)	0 (0,2)	0 (0, 0)	0.983
